# National Medical Association

**Published:** 1854-03

**Authors:** 


*National Medical Association.*

We hope our readers and the profession generally throughout the whole North-West, will remember that the next annual meeting of the American Medical Association will be held at St. Louis, commencing on the first *Tuesday* in *May.*

The time is near at hand. Delegates should be appointed by every regularly organized State, County and City Society. Each is entitled to one delegate for every *ten* of its members. Let such be selected as will be certain to attend. All the meetings of the Association heretofore have been highly interesting. Whether this one shall be equal in numbers and interest to any that have preceded it, depends much on the Profession in the West. Let every member of the Profession, then, feel a personal interest and pride in this matter.

